# The role of magnesium in the pathogenesis of osteoporosis

**DOI:** 10.3389/fendo.2024.1406248

**Published:** 2024-06-06

**Authors:** Lin Liu, Pan Luo, Pengfei Wen, Peng Xu

**Affiliations:** ^1^ Department of Joint Surgery, HongHui Hospital, Xi’an Jiaotong University, Xi’an, Shaanxi, China; ^2^ Department of Auricular Reconstruction, Plastic Surgery Hospital, Chinese Academy of Medical Sciences and Peking Union Medical College, Beijing, China

**Keywords:** magnesium, osteoporosis, parathyroid hormone, vitamin D, nutrition

## Abstract

Magnesium (Mg), a nutritional element which is essential for bone development and mineralization, has a role in the progression of osteoporosis. Osteoporosis is a multifactorial disease characterized by significant deterioration of bone microstructure and bone loss. Mg deficiency can affect bone structure in an indirect way through the two main regulators of calcium homeostasis (parathyroid hormone and vitamin D). In human osteoblasts (OBs), parathyroid hormone regulates the expression of receptor activator of nuclear factor-κ B ligand (RANKL) and osteoprotegerin (OPG) to affect osteoclast (OC) formation. In addition, Mg may also affect the vitamin D3 -mediated bone remodeling activity. vitamin D3 usually coordinates the activation of the OB and OC. The unbalanced activation OC leads to bone resorption. The RANK/RANKL/OPG axis is considered to be a key factor in the molecular mechanism of osteoporosis. Mg participates in the pathogenesis of osteoporosis by affecting the regulation of parathyroid hormone and vitamin D levels to affect the RANK/RANKL/OPG axis. Different factors affecting the axis and enhancing OC function led to bone loss and bone tissue microstructure damage, which leads to the occurrence of osteoporosis. Clinical research has shown that Mg supplementation can alleviate the symptoms of osteoporosis to some extent.

## 1 Introduction

Bone is continuously remodeled through the coordination and interaction between osteoclasts (OCs) and osteoblasts (OBs) to achieve bone homeostasis (1). Individuals with osteoporosis (OP), a systemic bone disease associated with ageing, are prone to fractures because of decreased bone density and quality caused by bone homeostasis imbalance and destruction of the bone microstructure (2, 3). In addition, patients with OP are more prone to micronutrient deficiency. An increase in micronutrient intake may have an osteoprotective effect on patients with OP (4). A meta-analysis by Feng et al. showed that dietary patterns were related to the incidence of OP (5). Magnesium (Mg), along with calcium (Ca) and vitamin D (VD), are key regulators of bone health and have an obvious influence on OP risk (6).

Approximately 99% of Mg is found in bones, muscles and soft tissues (7). Approximately 50–60% of Mg resides as surface substituents of the hydroxyapatite mineral component of bone (8). A considerable portion of skeletal Mg is mainly distributed on cortical bones (9). A large part of the Mg in bone may be deposited as apatite crystals. In addition to its structural function, Mg is a key element for all living cells, including OBs and OCs. In cells, Mg is essential for many physiological functions. First, Mg is the basis of ATP, and ATP is an essential energy source for cells (10). In addition, Mg is a cofactor of different enzymes associated with the synthesis of lipids and proteins. Moreover, Mg antagonizes Ca and acts as a signal sensor (11, 12). Therefore, changes in Mg homeostasis can affect cell and tissue function.

Many studies have suggested that Mg deficiency is a risk factor for OP (6, 13). In a study of rats, researchers have shown that controlling Mg intake in the daily diet can alleviate the symptoms of OP (13). If animals consume less Mg, it can lead to fragile bones and further cause microfractures of the trabeculae, resulting in extremely significant harm (14). Cohort research involving 73684 postmenopausal women revealed that lower Mg intake was related to reduced hip bone density (15). In addition, cross-sectional studies from the UK have found that dietary Mg may play a role in musculoskeletal health and is associated with population prevention strategies for myopenia, osteoporosis and fractures (16). Therefore, the main aim of this article is to summarize the effect of Mg on the pathogenesis of OP.

## 2 Pathogenesis of OP

Bone remodeling is regulated by osteocytes, OCs, OBs, bone lining cells, and endothelial cells in the bone microenvironment (17). These cells play a dynamic role in the formation and maintenance of bone integrity. Osteocytes exist in the voids of the matrix and are the mechanical receptors of bone tissue. Osteocytes maintain the physiological function of bone by interacting with various signals to transmit mechanical force to chemical signaling pathways (18). The OB plays an important role in body tissues and is synthesized by undifferentiated mesenchymal cells. Medical research has shown that OBs are involved in bone formation and growth. OCs are a type of multinucleated giant cell whose function is to promote bone resorption. Their main function is to absorb bone and prepare a matrix for bone generation (18, 19).

**Figure 1:**
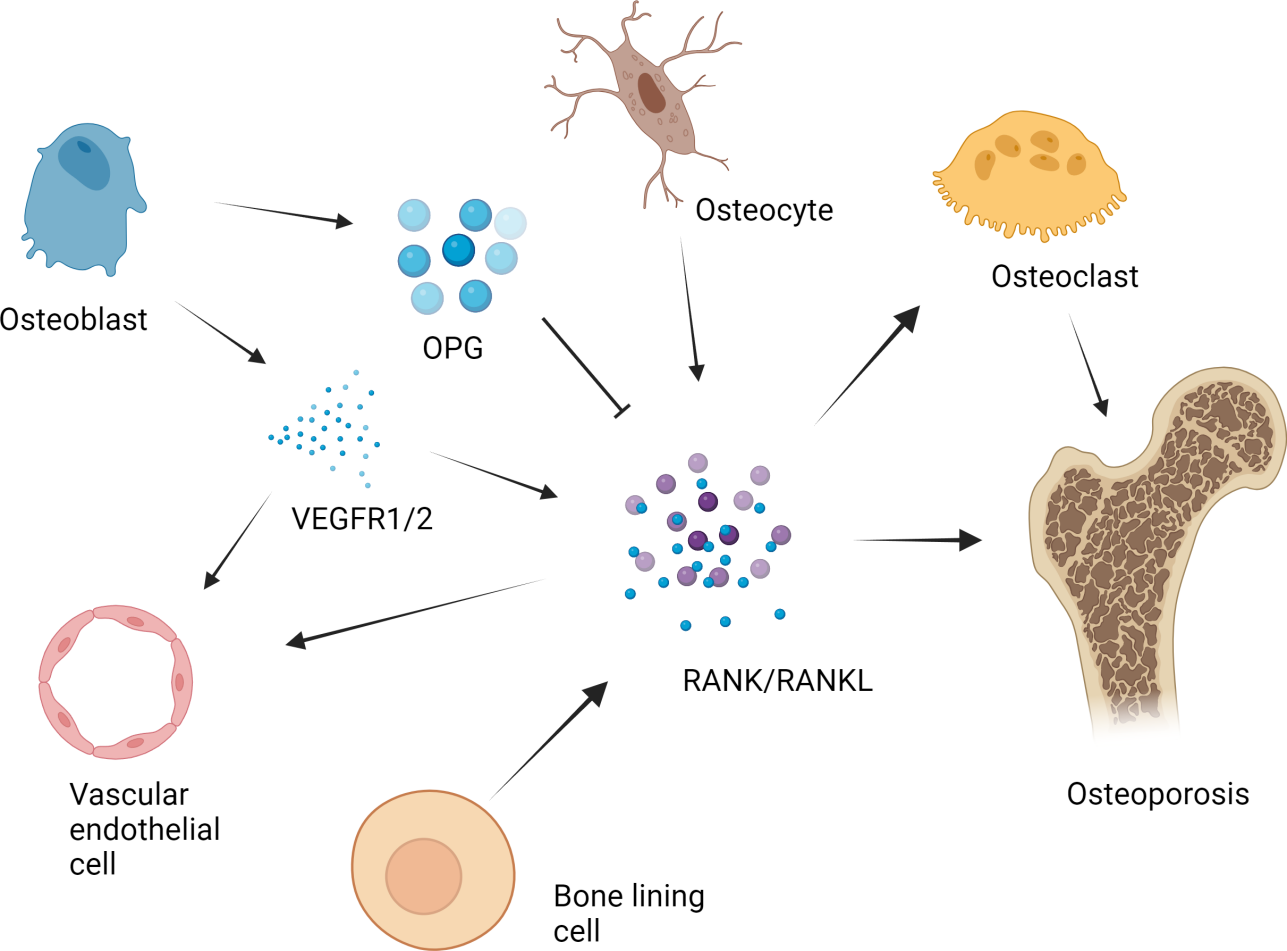
RANK/RANKL/OPG axis in the pathogenesis of osteoporosis. The RANK/RANKL/OPG axis plays a key role in the molecular mechanism of osteoporosis. Various factors affect the axis and cause osteoclast formation to a state of bone remodeling decompensation, resulting in a reduction in bone mass and damage to the bone tissue microstructure, which leads to the occurrence of osteoporosis. For example, in the process of osteoclast differentiation and activation, osteoblasts express RANKL and OPG, which participate in the regulation of osteoclast differentiation. RANKL binds to RANK and activates the downstream signaling pathway, leading to the activation of osteoclast differentiation. OPG suppresses the above effects by inhibiting the RANKL-RANK interaction. The RANKL secreted by osteocytes plays an important role in the formation of osteoclasts in bone. The expression of RANK in vascular endothelial cells in the bone microenvironment is upregulated by vascular endothelial growth factor (VEGF), which enhances the angiogenic response to RANKL. Previous studies have shown that bone lining cells express RANKL and other osteoblast markers during active remodeling intervals in the lining, which is responsible for the interaction between RANKL and the osteoclast precursor receptor RANK.

A variety of proteins and signaling molecules are involved in the regulation of bone homeostasis. It is wildly believed that the RANK/RANKL/OPG axis is a key factor in the molecular mechanism of OP (20–22). (**Figure 1**) Various factors affect the axis and enhance the formation of OCs to a state of decompensation, resulting in reduced bone mass and damage to the bone tissue microstructure, which leads to the occurrence of OP (23). In the process of OC differentiation and activation, OBs participate in the regulation of OC differentiation by expressing RANKL and OPG (24). RANKL binds to RANK and activates OC differentiation through the activation of downstream signaling pathways, while OPG inhibits these effects by inhibiting the RANKL-RANK interaction.

Osteocytes have been shown to be regulators of mineral metabolism and periluminal matrix remodeling as well as the function of mechanosensory cells (25). It has been determined that osteocytes express RANKL and that RANKL secreted by osteocytes is most important for the formation of physiologically supported OCs in developing bones (26). After long-term research, some scholars have shown that bone lining cells can synthesize factors such as RANKL (27). Further research revealed that these cells can also regulate the ability of RANKL to bind to RANK receptors (28). Long-term studies have shown that vascular endothelial growth factor (VEGF) is involved in bone remodeling (29). OBs can express VEGF receptor 1 (VEGFR1) and VEGFR2 and release VEGF upon stimulation with VD3 (30). The expression of RANK on endothelial cells in the bone microenvironment was upregulated by VEGF, which subsequently enhanced the angiogenic response to RANKL (29).

The most common cause of OP is a lack of estrogen in postmenopausal women, which can lead to increased OC activity and bone mass loss, resulting in OP and osteoporotic fractures (31). Ca is the most basic mineral component in bone, and insufficient Ca intake will lead to decreases in bone mass. Therefore, VD deficiency can cause OP. Parathyroid hormone (PTH) is a hormone secreted by the parathyroid gland that is mainly responsible for the metabolism of Ca and phosphorus and regulating the levels of these two elements in the body. PTH plays a key role in maintaining Ca and phosphorus levels. A high or low level of secreted PTH may lead to abnormal metabolism of the two elements in the body, leading to OP (32).

## 3 The role of Mg in the pathogenesis of OP

Mg can strongly promote bone development and mineralization, by increasing the activity of phosphatase (33). Insufficient intake of magnesium in daily diet can lead to a decrease in bone mineral density. According to the results of animal studies, insufficient dietary Mg intake promotes the occurrence of OP (6). That not only reduces bone density but also alters the levels of PTH and 1,25(OH)2-VD in tissues, thereby inhibiting the body’s absorption of Ca and ultimately inducing hypocalcemia. Scholars have conducted long-term studies on humans, and the results show that hypomagnesemia can significantly inhibit the synthesis of PTH in the body, leading to damage to related organs (34). For example, clinical studies by Ohya et al. have shown a significant correlation between serum Mg and intact PTH (iPTH) levels (35). The clinical evidence of Cheung et al. shows that combined Mg and VD therapy may increase serum 25-hydroxyvitamin D concentration more effectively than supplementation of VD alone (36). Due to the increase in PTH levels, the activity of adenylate cyclase can be enhanced, thereby promoting the secretion of cyclic adenosine monophosphate (AMP) (13, 37). And these enzymes need sufficient participation of Mg to function, which means that resistance to PTH can lead to not fully stimulated enzyme activity. Notably, hypomagnesemia can also induce an inflammatory response (38) and further lead to bone loss (39). Scholars have analyzed the effects of Mg on blood vessels by constructing animal models and concluded that Mg can improve endothelial function and lower blood pressure. However, a decrease in the volume of blood vessels within the bone may trigger nerve injury-induced OP (40) and OP in elderly individuals (41).

### 3.1 Effect of Mg on OBs in the pathogenesis of OP

Several researchers have investigated the influence of Mg on the differentiation or function of OBs (42, 43). Bed et al. indicated that extracellular Mg (2+) and melastatin-like transient receptor potential 7 (TRPM7) are important for platelet-derived growth factor (PDGF)-induced proliferation and migration of human osteoblasts (44).

**Figure 2:**
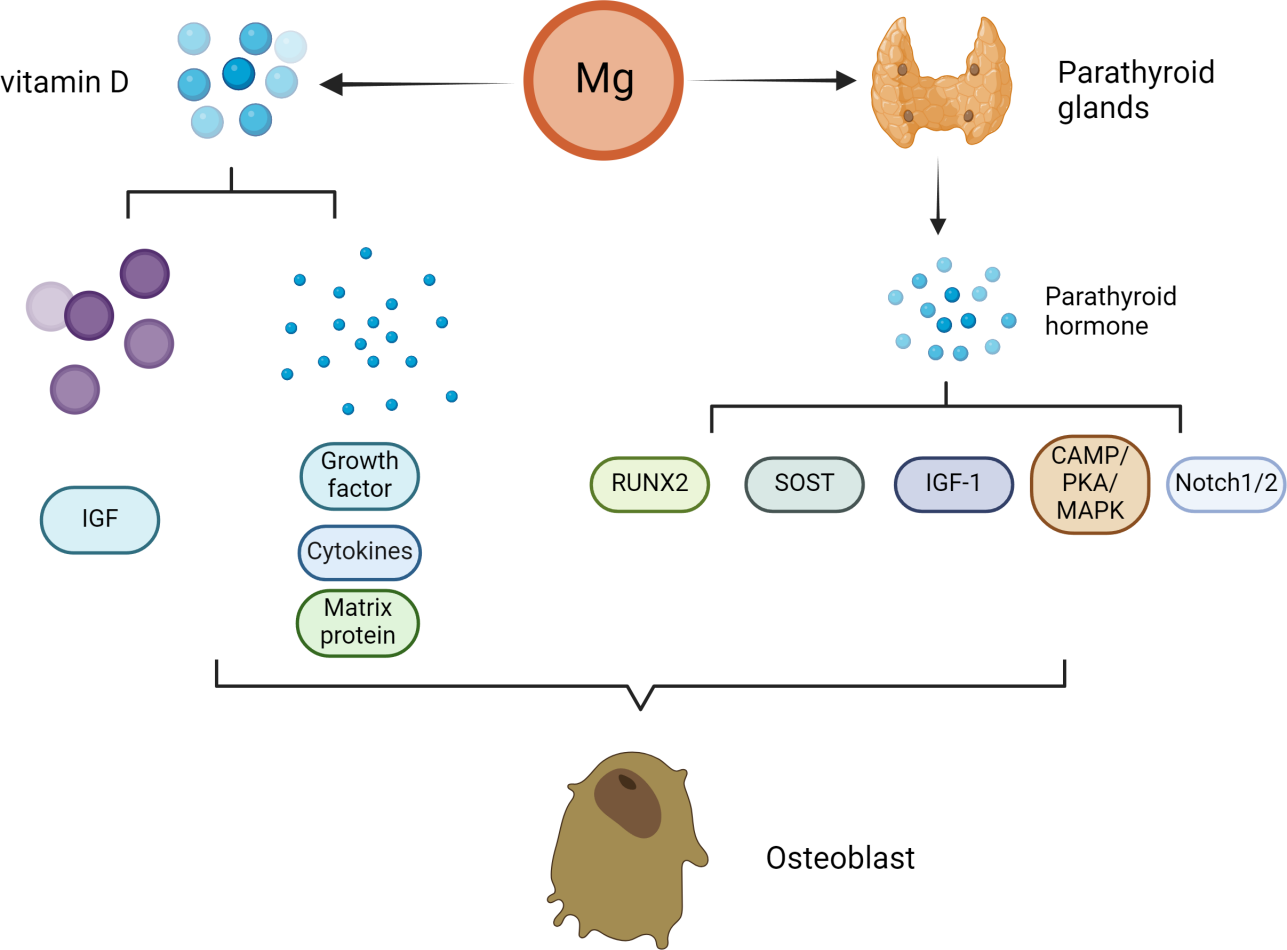
Effect of Mg on osteoclasts in the pathogenesis of osteoporosis. With decreasing serum Mg, the serum parathyroid hormone level increased. PTH enhances bone formation through a variety of mechanisms, including direct action on osteoblasts, induction of insulin-like growth factor (IGF)-1 and possible inhibition of sclerostin (SOST). The direct stimulation of osteoblast function by PTH is mainly mediated by the activation of cyclic adenosine monophosphate (cAMP)/protein kinase A (PKA) and mitogen activated protein kinase (MAPK). PTH signal transduction in osteoblasts affects Runx2. Runx2 is a transcription factor that plays an important role in osteoblast differentiation and function. Notch signal regulation is another mechanism by which PTH promotes osteogenesis. Notch1 inhibits the differentiation of mesenchymal progenitor cells and mature osteoblasts and bone trabecular formation, and Notch2 has a similar inhibitory effect on the function of osteoblasts. In addition to patients with parathyroid hormone secretion disorders, patients with Mg deficiency usually have low serum vitamin D concentrations. Vitamin D can affect not only the proliferation of osteoblasts but also the differentiation of osteoblasts. The extracellular environment (growth factors, cytokines, matrix proteins, calcium/phosphorus and other signaling molecules) and the intracellular environment (e.g., insulin-like growth factor binding protein-6) influence the final effect of vitamin D.

*In vivo* experiments and clinical studies have shown that high concentrations of Mg can inhibit the secretion of PTH (45–47). Under normal physiological conditions, Mg affects the secretion of PTH in a manner similar to that of Ca. (**Figure 2**) Specifically, increased serum Mg binds to Ca sensor receptors on parathyroid cells, resulting in increased levels of intracellular Ca and decreased PTH secretion. In contrast, the level of serum PTH increased with decreasing serum Mg. PTH enhances bone formation via different mechanisms, including direct activation on OBs, induction of insulin-like growth factor (IGF)-1 and possible inhibition of sclerostin (SOST) (48). PTH mainly promotes OB division through the involvement of enzymes such as protein kinase A (PKA) (49). Scholars have explored the effects of PTH on bone tissue by constructing animal models and conducting intergroup control experiments in humans (50). The results showed that PTH administration can significantly promote the division of OBs, thereby accelerating the formation of bone tissue stimulated. Moreover, PTH administration can also promote the deposition of mineralized matrix by regulating the proliferation of osteoblast precursors and other pathways (51).

Nevertheless, the main direct effect of iPTH on OBs *in vivo* is to reduce the apoptosis of OBs but not to promote the proliferation of preosteoblasts. Moreover, PTH signal transduction in OBs affects Runx2, which is a transcription factor associated with OB differentiation and function (52). In addition to Runx2, many studies have suggested that multiple additional target genes (liver ligand protein B2, IGF-1, FGF2, PTH-related protein and MMP13) in OBs contribute to the antiapoptotic effect of iPTH therapy (53–57). In addition to apoptosis, iPTH can play a vital role in OB differentiation and function through the WNT signaling pathway (58). However, WNT signaling has different effects on OBs depending on their differentiation stage (59). Research has shown that in preosteoblasts, WNT signaling can stimulate and accelerate the division of OBs, playing a very important role in the development of bone tissue (60). In mature OBs, these signals can increase the level of OPG, which is the bait receptor for RANKL, thereby slowing the absorption of bone (61).

PTH can also promote bone tissue growth through Notch signaling. Previous studies have shown that PTH upregulates Jag1 expression in OBs (62). In addition to *Jag1*, PTH can up-regulate the expression of other Notch components in bone, especially *Notch2* and *Dll1* (63). Researchers have shown that Notch1 can prevent the proliferation and division of mature OBs and interfere with the formation of bone trabeculae. Moreover, Notch2 has a similar inverse effect on OB function (64). Studies by Canalis et al. have shown that gene activation of Notch2 signal transduction stimulates OC differentiation and absorption (65). In addition, Jesus et al. shown that the increases in Notch ligands and receptors lead to Notch activation, as PTH also elevated the expression of several Notch target genes in bone, particularly Hes1 that was elevated across all the experiments (63). In addition, the activation of WNT signal in OBs or osteocytes increases the Notch signal in bone, indicating that there is crosstalk between the two pathways (66, 67).

In addition to PTH secretion disorders, the serum concentrations of the VD active metabolite 1,25(OH) 2 D 3 are often low in patients with Mg deficiency. This may be due explained by low serum PTH levels or renal are resistant to PTH because PTH is the main physiological regulator of 1,25(OH)_2_-VD synthesis. Mg deficiency is harmful to this process because the synthesis of 1,25(OH)_2_-VD depends on the presence of Mg. Previous studies have demonstrated the direct influence of 1α,25(OH) (2)D(3) on the survival of OBs, which varies with the treatment time, dose, source and environment of the OBs (68–70).

Additionally, 1α,25(OH) (2)D(3) can affect not only the proliferation of OBs but also the differentiation of OBs (71, 72). The extracellular environment (VEGF, cytokines, Ca/phosphorus ions et al.) and the intracellular environment (IGF binding protein-6) affect the final effect of 1α,25(OH) (2)D(3) (73). These factors can regulate the role of 1α,25(OH) (2)D(3) and impact the final reaction. A typical intracellular pathway is WNT signaling. Standard WNT signals are essential for bone formation. The lipoprotein-related receptors 5 and 6 (LRP5/6) promote the secretion of members of the WNT-related protein family and binds to the membrane receptor on OBs. The absence of LRP5 leads to a decrease in the number of OBs, delayed mineralization and a reduction in peak bone mineral density. However, 1α,25(OH) (2)D(3) can cause the binding of vitamin D receptor (VDR) to the LRP5 locus (74). Therefore, VD3 is a key factor in OB differentiation and bone generation because it affects WNT signaling (74, 75).

### 3.2 Effect of Mg on OC in the pathogenesis of OP

Mg deficiency promotes OC formation and bone loss (6, 76). Mg deficiency in animal models has been shown to stimulate the generation of cytokines, which can promote bone resorption by OCs. For example, an increase in RANKL and a reduction in OPG can lead to increased bone resorption (77).

**Figure 3:**
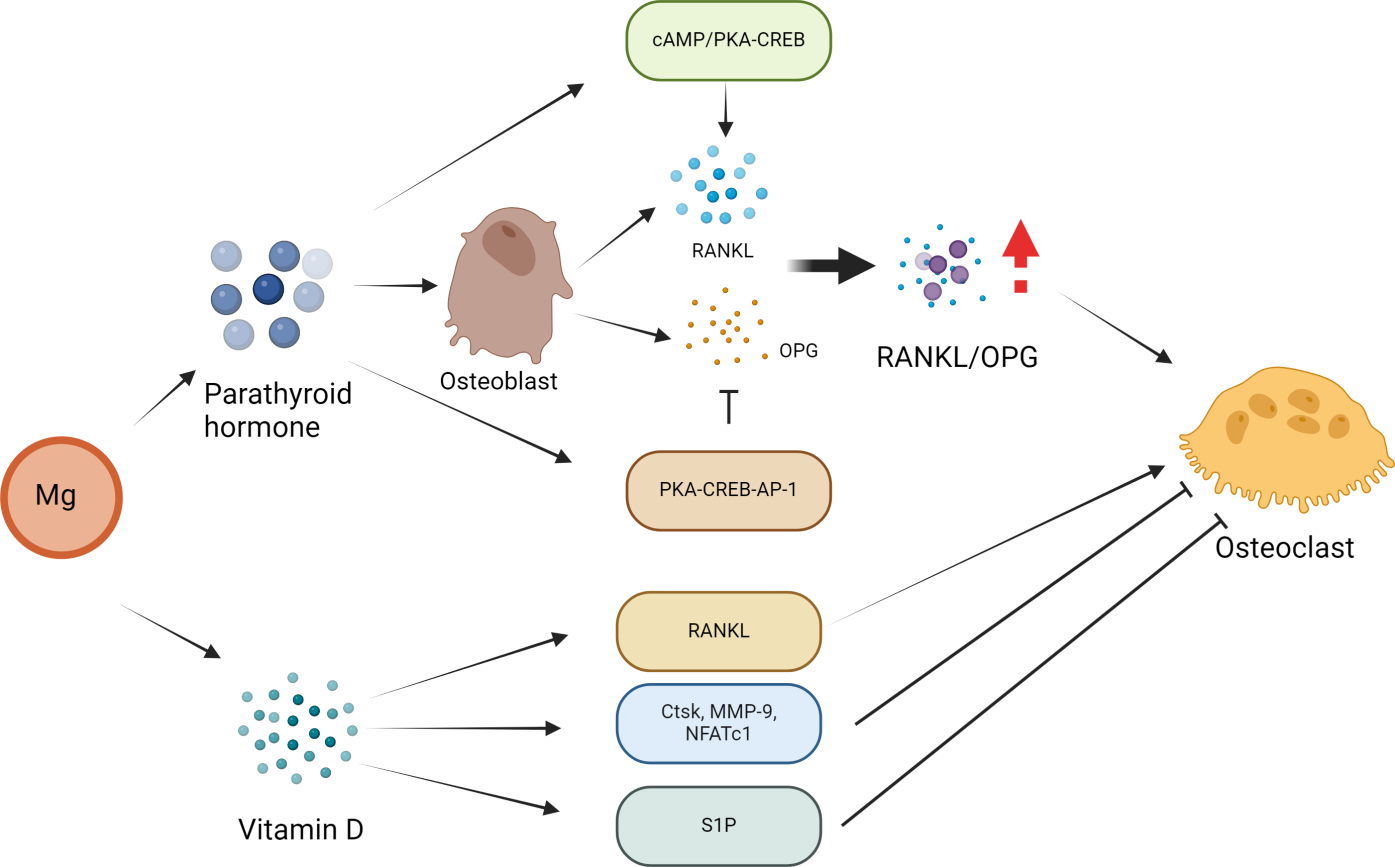
Effect of Mg on osteoclasts in the pathogenesis of osteoporosis. PTH may activate osteoclasts indirectly through its effect on osteoblasts, thus inducing bone resorption. In osteoblasts, parathyroid hormone regulates the expression of RANKL and OPG, and these two receptors play a leading role in osteoclast formation. PTH directly increases the expression of RANKL by activating the cAMP/PKA-CREB pathway and inhibits the expression of OPG via the PKA-CREB-AP-1 pathway. These PTH actions lead to an increase in the RANKL/OPG ratio, which is considered to be the main mechanism by which PTH affects osteoclast formation and bone resorption. The formation of OCs can also be regulated directly by vitamin D. In addition, vitamin D inhibits the expression of OC-related genes and proteins (Ctsk, MMP-9 and NFATc1) to inhibit osteoclast formation. In addition, vitamin D and its analogue decalcified alcohol inhibit OC formation by regulating the sphingosine-1-phosphate (S1P) receptor system.

As previously described, high levels of Mg inhibit PTH secretion (45). In body tissue, PTH mainly enhances bone resorption by enhancing OC activity (78). In recent years, some scholars have conducted *in vitro* experiments and found that if OCs are cocultured with conditioned medium from stromal cells and other similar cells, they are more likely to interact with PTH (79). Therefore, PTH may indirectly activate OCs via its effect on OBs, thus inducing bone resorption. In OBs, RANKL and OPG play different roles by coordinating with each other to maintain the stability of bone tissue (80, 81). The combination of RANKL and RANK can accelerate the generation of OCs. The above functions of RANKL are inhibited by OPG, thereby reducing the activity of OCs (80, 81). (**Figure 3**) Specifically, with the participation of OPG, RANKL binding can inhibit its entry into the corresponding receptor (82). After PTH administration, OPG mRNA (83) can be detected in rat bones. Currently, Fu et al. (84) conducted a series of *in vitro* studies to explore the mechanism of action of PTH. These results indicate that PTH mainly enhances RANKL levels by activating cAMP/PKA-CREB. and during this process, it can also downregulate OPG levels by regulating PKA-CREB-AP-1.

Mg may affect the bone remodeling activity of VD3. For example, VD3 usually participate the activation of OBs and OCs. Mg deficiency leads to an imbalance in OC activation, which in turn leads to bone resorption (85). A Mg-deficient diet may be associated with abnormal bone remodeling, and increased OC activity as well as the risk of OP in animals. In addition, an Mg-integrated diet results in Ca deposition in bones through interactions with VD3, thus increasing bone mass to block or limit OP (86). In addition, 1α,25(OH) (2)D(3) can indirectly promote OC formation by enhancing the level of RANKL. The formation of OCs is also associated with 1α,25(OH) (2)D (3). In addition, 1α,25(OH) (2)D (3) prevents the expression of OC-associated proteins, such as *Ctsk, MMP-9* and *NFATc1* (87). Sakai reported that 1α,25(OH) (2)D (3) inhibits OC generation by preventing the expression of *c-Fos* and *NFATc1 (* 88). Kikuta et al. reported that 1α,25(OH) (2)D(3) promoted OC migration by regulating the sphingosine 1-phosphate (S1P) receptor system (89). Therefore, 1α,25(OH) (2)D (3) can promote the genealogical nature of OCs and inhibit the formation of OCs in different environments, thus achieving bone health balance.

### 3.3 Effect of Mg on osteocytes in the pathogenesis of OP

Recent evidence suggests that osteocytes are important cellular targets for PTH (90). PTH can prevent the synthesis of sclerotin in osteocytes to accelerate the development of bone tissue. Research has shown that SOST is a secreted protein that can directly act on the WNT–β-catenin signaling pathway to delay the development of bone tissue (91). Scholars have also pointed out that the substances formed by the binding of WNT ligands to frizzled (Fzd) receptors can act on the WNT–β-catenin signaling pathway to accelerate the development of bone tissue (50). The relevant research results indicate that RANKL can also be produced by osteocytes (26). In osteocytes, this protein is upregulated by PTH, which in turn plays a role in osteoclastogenesis (92). Therefore, Mg can regulate PTH signals through paracrine mediators (such as RANKL) in osteocytes to affect bone remodeling.

Mg is the second most abundant cation in cells and is an essential factor in the synthesis and metabolism of PTH and VD (93, 94). Previous studies have shown that the activities of the three main enzymes that determine 25(OH)D levels and VD-binding proteins are dependent on Mg (95). In addition, a previous study showed that people with a combined 25(OH)D and Mg deficiency were more likely to have OP than those with a single 25(OH)D deficiency (96). These findings suggest a potential interaction between VD and Mg. The osteocytes are closely related to increased blood phosphate and 1,25(OH)2 VD (97, 98). Pereira et al. reported that the overall influence of active VD sterols on bone in patients with chronic kidney disease is to promote the generation of osteocytes in the early stage of maturation, increase the number of late osteocytes, and increase osteocyte turnover (97). In addition, VD may be a key factor in regulating OB/OC/osteocyte coupling by promoting RANKL/OPG expression (97).

### 3.4 Effect of Mg on vascular endothelial cells in the pathogenesis of OP

Bone angiogenesis is closely associated with bone metabolism, remodeling and repair. There is evidence that the influence of PTH is mediated by the BMP/Smad1 pathway and is associated with the regulation of angiogenesis (99). VEGF can increase bone vascular invasion and regulate the morphology of growth plates (100). Fu et al. reported that the zinc-finger E-box-binding homeobox-1 (ZEB1)/Notch signaling pathway controls the recruitment/differentiation of perivascular bone progenitor cells, thus promoting osteogenesis by regulating the expression of many vascular secretory factors (such as TGF-β 1, 2, BMP2, BMP4, FGF1 and Noggin) (101). All these vascular secretory factors are thought to be actively involved in osteogenesis.

As described in previous studies, Mg deficiency is associated with decreased PTH levels and induced PTH resistance in terminal organs (102). Currently, VEGF is the most well-studied angiogenic factor in the skeletal system of mammals. As the most important HIF1α target gene after translocation to the nucleus, VEGF is considered to be a key link between angiogenesis and osteogenesis (100, 103, 104). Many scholars have shown that PTH may increase the expression of VEGF in OBs, and VEGF can increase the activity of endothelial cells and promote angiogenesis (99, 105–107). In addition, Ding et al. showed that PTH may affect the expression of VEGF through the PKA/pAKT/HIF1α pathway, thus affecting angiogenesis (108). In addition, Previous studies have suggested that PTH can induce oxidative stress, but current studies have shown that its effect depends on the level of PTHR in endothelial cells (109).

### 3.5 Effect of Mg on osteosarcopenia

Mg plays an important metabolic and physiological role in musculoskeletal system (110, 111). For example, the results of Welch et al. show that dietary Mg may have a clinical effect on skeletal muscle and bone health in the middle-aged and elderly (16). In addition, in a nationwide cross-sectional survey in 10,279 participants with hypertension based on NHANES, a positive association was observed between dietary Mg intake and appendicular skeletal muscle mass index (ASMI), but not between Mg supplements and ASMI, implying the importance and uniqueness of dietary source of Mg (112). The positive correlation between dietary Mg intake and ASMI can be explained by several possibilities. Animal studies have shown that Mg may improve exercise performance by increasing glucose availability in muscles and blood (113). Mg may also affect muscle performance by maintaining protein synthesis and circulation in muscle through energy metabolism (114). In addition, recent studies have shown that Mg deficiency may increase inflammation and be associated with muscle damage. Inflammation is one of the important factors leading to the occurrence and development of myopenia (115). Experimental animals with Mg deficiency showed systemic inflammation and increased levels of inflammatory markers. Dietary Mg supplements reduced the production of pro-inflammatory cytokines and oxidative stress (116). Several studies have shown that higher Mg intake is associated with lower serum CRP (117).

Previous studies showed that a decrease in muscle mass may be the result of a decrease in protein synthesis or an increase in protein degradation, particularly the ATP-dependent ubiquitin–proteasome proteolytic pathway (118–120). Mg may also affect muscle performance through energy metabolism (production of ATP), transmembrane transport, and muscle contraction and relaxation (121). Skeletal muscle aging is strongly affected by the loss of balance between molecular and muscle cell injury and repair processes, and is characterized by immune activation (122). Chronic systemic inflammation during aging is associated with muscle decrease and weakness, and involves the increase of resident macrophages in aging muscles (123). Cui et al. found that oral Mg supplements could modulate macrophage phenotype by decreasing the M2 population and reduce inflammation during sarcopenia (124). In addition, they found that oral Mg supplements can reduce the deterioration of muscle function in the later stage of muscular dystrophy (124).

## 4 Mg supplements and clinical treatment of osteoporosis

An increasing number of clinical studies are exploring the influence of Mg supplementation on bone mass and OP. Consistent with the results of a meta-analysis by Farsinejad-Marj et al. (125), Groenendijk et al. reported a significant positive correlation between Mg intake and hip joint BMD (126). Moreover, Groenendijk et al. found a significant positive correlation between Mg intake and the bone density of the femoral neck but no significant association between Mg intake and the bone density of the lumbar spine. (**Table 1**) In addition, Aydin et al. analyzed the influence of various nutrients on OP (127). Researchers have studied the effects of bone Mg supplementation on postmenopausal women. The experimental group received daily oral administration of Mg citrate, while the control group did not receive any form of intervention. Afterwards, blood was collected for testing, and the results showed a significant decrease in PTH content in the experimental group; moreover, the content of osteocalcin increased (127).

**Table 1:** Clinical evaluation of magnesium in the treatment of osteoporosis.

First Author, year	Country	Participants	Case	Mean Mg intake	Sources	Relevant Outcomes	Reference
Inge Groenendijk, 2022	Netherlands	Men and woman≥60 y	988	Men:350mg/day Woman:300mg/day	Food and Supplement	BMC/TB BMD/Hip BMD/FN BMD/LS BMD/BTM/Fracture risk	(126)
Hasan Aydin, 2010	Turkey	PM women	20	1830 mg/day	Supplement	FN BMD/LS BMD	(127)
G Stendig-Lindberg, 1993	Israel	PM women 61.2± 6.2 y	31	250–750 mg/day	Supplement	TB BMD	(128)
Liam E.Fouhy, 2023	United States	Men and woman 47–79 y	955	704 ± 153 mg/day	Food	BMD	(129)
Nicola Veronese, 2017	United States	Men and woman 60.6 ± 9.1 y	3765	Men: 205/269/323/398mg/day Woman: 190/251/306/373mg/day	Food and Supplement	Fracture risk	(130)
Kathryn M Ryder, 2005	United States	Black/White men and woman 70–79 y	238	Black Women 279.2 ± 115.6; Black Men 304.7 ± 127.5; White Women 307.6 ± 121.9; White Men 330.8 ± 111.9;	Food and Supplement	TH BMD	(131)

Mg, magnesium; BMC, bone mineral content; BMD, bone mineral density; FN, femoral neck; TB, total body; LS, lumbar spine; BTM, bone turnover markers; PM, postmenopausal.

A previous Israeli clinical case−control study showed that Mg therapy significantly increased bone mineral density in 71% of women and prevented bone loss in 16% of women (128). In addition, Mg supplements in menopausal women have been shown to be more effective in combination with other elements. For example, patients who consumed a complete supplement of 500 mg of Ca citrate and 200 mg of Mg oxide exhibited an increase in the average bone mineral density of 11% compared with that of patients who received 500 mg of Ca citrate alone (132). Ca: Mg intake in the range of 2.2–3.2 seems to have the most protective effect, which indicates that the balance of these nutrients can be included in the recommendations for patients with OP (129). Veronese et al. reported that Mg had a greater effect on fracture risk in women than in men (62% and 53%, respectively) (130). Ryder et al. also reported that the correlation between Mg intake and whole-body BMD and hip BMD in men was lower than that in women (131).

A large number of studies have indicated that moderate supplementation of Ca and VD is an effective intervention for preventing bone loss (133). Some scholars believe that supplementing 1000 mg/d Ca and VD appropriately is necessary to prevent bone loss in individuals in the elderly population (134, 135). In addition, the intake of Mg should also be strictly controlled, with adult men consuming approximately 350 milligrams per day and women consuming 300 milligrams per day (136). Not all older people can meet this intake recommendation. In Western countries, the average Mg intake of healthy elderly people ranges from 274 to 421 mg/day for men and from 227 to 373 mg/day for women. This proportion is lower among the weaker elderly. Dietary sources rich in Mg include leafy vegetables, legumes, nuts, and seeds (137, 138). In addition, nuts and seeds contain abundant protein and Ca, which has a positive impact on bone homeostasis. These phenomena indicate that the effect of nutrients on OP cannot be considered in isolation.

## 5 Conclusion and prospects

There is an important relationship between the occurrence and development of OP and the imbalance of bone homeostasis, in which the interaction among OBs, OCs and osteocytes is a key factor in maintaining bone stability. Although the effects of various trace elements on bone health and OP have been widely studied, there are no related articles summarizing the role of Mg in maintaining bone homeostasis. Therefore, in the present article, we explore the influence of Mg on various cells involved in the maintenance of bone homeostasis during the pathogenesis of OP and summarize the current clinical studies on the use of Mg in the treatment of OP. The findings are expected to fully clarify the relationship between Mg and OP from a basic to a clinical perspective.

## Author contributions

LL: Conceptualization, Data curation, Formal analysis, Investigation, Resources, Software, Validation, Visualization, Writing – original draft, Writing – review & editing. PL: Conceptualization, Formal analysis, Funding acquisition, Investigation, Project administration, Supervision, Visualization, Writing – original draft, Writing – review & editing. PW: Conceptualization, Data curation, Formal analysis, Investigation, Methodology, Project administration, Supervision, Writing – original draft, Writing – review & editing. PX: Conceptualization, Funding acquisition, Investigation, Methodology, Project administration, Software, Validation, Writing – original draft, Writing – review & editing.

## References

[1] Nieves JW. Osteoporosis: the role of micronutrients. Am J Clin Nutr. (2005) 81:1232s–9s. doi: 10.1093/ajcn/81.5.1232 15883457

[2] Adejuyigbe B Kallini J Chiou D Kallini JR. Osteoporosis: molecular pathology, diagnostics, and therapeutics. Int J Mol Sci. (2023) 24. doi: 10.3390/ijms241914583 PMC10572718 37834025

[3] Ström O Borgström F Kanis JA Compston J Cooper C McCloskey EV. Osteoporosis: burden, health care provision and opportunities in the eu: A report prepared in collaboration with the international osteoporosis foundation (Iof) and the european federation of pharmaceutical industry associations (Efpia). Arch osteoporosis. (2011) 6:59–155. doi: 10.1007/s11657-011-0060-1 22886101

[4] Stazi AV. [Micronutrient deficiencies in osteoporosis]. Minerva Med. (2013) 104:455–70. 24008608

[5] Feng W Wang X Huang D Lu A. Role of diet in osteoporosis incidence: umbrella review of meta-analyses of prospective observational studies. Crit Rev Food Sci Nutr. (2023) 63:3420–9. doi: 10.1080/10408398.2021.1989374 34644187

[6] Castiglioni S Cazzaniga A Albisetti W Maier JA. Magnesium and osteoporosis: current state of knowledge and future research directions. Nutrients. (2013) 5:3022–33. doi: 10.3390/nu5083022 PMC3775240 23912329

[7] Jahnen-Dechent W Ketteler M. Magnesium basics. Clin Kidney J. (2012) 5:i3–i14. doi: 10.1093/ndtplus/sfr163 26069819 PMC4455825

[8] Fiorentini D Cappadone C Farruggia G Prata C. Magnesium: biochemistry, nutrition, detection, and social impact of diseases linked to its deficiency. Nutrients. (2021) 13. doi: 10.3390/nu13041136 PMC8065437 33808247

[9] Alfrey AC Miller NL. Bone magnesium pools in uremia. J Clin Invest. (1973) 52:3019–27. doi: 10.1172/jci107500 PMC302576 4584344

[10] Kleczkowski LA Igamberdiev AU. Magnesium and cell energetics: at the junction of metabolism of adenylate and non-adenylate nucleotides. J Plant Physiol. (2023) 280:153901. doi: 10.1016/j.jplph.2022.153901 36549033

[11] Iseri LT French JH. Magnesium: nature's physiologic calcium blocker. Am Heart J. (1984) 108:188–93. doi: 10.1016/0002-8703(84)90572-6 6375330

[12] Li FY Chaigne-Delalande B Kanellopoulou C Davis JC Matthews HF Douek DC. Second messenger role for mg2+ Revealed by human T-cell immunodeficiency. Nature. (2011) 475:471–6. doi: 10.1038/nature10246 PMC3159560 21796205

[13] Rude RK Singer FR Gruber HE. Skeletal and hormonal effects of magnesium deficiency. J Am Coll Nutr. (2009) 28:131–41. doi: 10.1080/07315724.2009.10719764 19828898

[14] Boskey AL Rimnac CM Bansal M Federman M Lian J Boyan BD. Effect of short-term hypomagnesemia on the chemical and mechanical properties of rat bone. J orthopaedic research: Off Publ Orthopaedic Res Soc. (1992) 10:774–83. doi: 10.1002/jor.1100100605 1403290

[15] Orchard TS Larson JC Alghothani N Bout-Tabaku S Cauley JA Chen Z. Magnesium intake, bone mineral density, and fractures: results from the women's health initiative observational study. Am J Clin Nutr. (2014) 99:926–33. doi: 10.3945/ajcn.113.067488 PMC3953885 24500155

[16] Welch AA Skinner J Hickson M. Dietary magnesium may be protective for aging of bone and skeletal muscle in middle and younger older age men and women: cross-sectional findings from the uk biobank cohort. Nutrients. (2017) 9:1189. doi: 10.3390/nu9111189 29084183 PMC5707661

[17] Ansari N Sims NA. The cells of bone and their interactions. Handb Exp Pharmacol. (2020) 262:1–25. doi: 10.1007/164_2019_343 32006260

[18] Gao Y Patil S Jia J. The development of molecular biology of osteoporosis. Int J Mol Sci. (2021) 22. doi: 10.3390/ijms22158182 PMC8347149 34360948

[19] Aibar-Almazán A Voltes-Martínez A Castellote-Caballero Y Afanador-Restrepo DF Carcelén-Fraile MDC López-Ruiz E. Current status of the diagnosis and management of osteoporosis. Int J Mol Sci. (2022) 23. doi: 10.3390/ijms23169465 PMC9408932 36012730

[20] Ding Z Shi H Yang W. Osteoprotective effect of cimiracemate in glucocorticoid-induced osteoporosis by osteoprotegerin/receptor activator of nuclear factor K B/receptor activator of nuclear factor kappa-B Ligand signaling. Pharmacology. (2019) 103:163–72. doi: 10.1159/000495509 30695776

[21] Lee SJ Jang SA Kim SC Ryuk JA Ha H. Lophatherum gracile bronghiart suppresses receptor activator of nuclear factor kappa-B ligand-stimulated osteoclastogenesis and prevents ovariectomy-induced osteoporosis. Int J Mol Sci. (2022) 23. doi: 10.3390/ijms232213942 PMC9699449 36430416

[22] Ferrari S Langdahl B. Mechanisms underlying the long-term and withdrawal effects of denosumab therapy on bone. Nat Rev Rheumatol. (2023) 19:307–17. doi: 10.1038/s41584-023-00935-3 37024711

[23] Boyce BF Li J Yao Z Xing L. Nuclear factor-kappa B regulation of osteoclastogenesis and osteoblastogenesis. Endocrinol Metab (Seoul Korea). (2023) 38:504–21. doi: 10.3803/EnM.2023.501 PMC10613774 37749800

[24] Zhivodernikov IV Kirichenko TV Markina YV Postnov AY Markin AM. Molecular and cellular mechanisms of osteoporosis. Int J Mol Sci. (2023) 24. doi: 10.3390/ijms242115772 PMC10648156 37958752

[25] Bonewald LF. The amazing osteocyte. J Bone mineral research: Off J Am Soc Bone Mineral Res. (2011) 26:229–38. doi: 10.1002/jbmr.320 PMC3179345 21254230

[26] Nakashima T Hayashi M Fukunaga T Kurata K Oh-Hora M Feng JQ. Evidence for osteocyte regulation of bone homeostasis through rankl expression. Nat Med. (2011) 17:1231–4. doi: 10.1038/nm.2452 21909105

[27] Streicher C Heyny A Andrukhova O Haigl B Slavic S Schüler C. Estrogen regulates bone turnover by targeting rankl expression in bone lining cells. Sci Rep. (2017) 7:6460. doi: 10.1038/s41598-017-06614-0 28744019 PMC5527119

[28] Hauge EM Qvesel D Eriksen EF Mosekilde L Melsen F. Cancellous bone remodeling occurs in specialized compartments lined by cells expressing osteoblastic markers. J Bone mineral research: Off J Am Soc Bone Mineral Res. (2001) 16:1575–82. doi: 10.1359/jbmr.2001.16.9.1575 11547826

[29] Deckers MM Karperien M van der Bent C Yamashita T Papapoulos SE Löwik CW. Expression of vascular endothelial growth factors and their receptors during osteoblast differentiation. Endocrinology. (2000) 141:1667–74. doi: 10.1210/endo.141.5.7458 10803575

[30] Engsig MT Chen QJ Vu TH Pedersen AC Therkidsen B Lund LR. Matrix metalloproteinase 9 and vascular endothelial growth factor are essential for osteoclast recruitment into developing long bones. J Cell Biol. (2000) 151:879–89. doi: 10.1083/jcb.151.4.879 PMC2169432 11076971

[31] Cheng CH Chen LR Chen KH. Osteoporosis due to hormone imbalance: an overview of the effects of estrogen deficiency and glucocorticoid overuse on bone turnover. Int J Mol Sci. (2022) 23. doi: 10.3390/ijms23031376 PMC8836058 35163300

[32] Neer RM Arnaud CD Zanchetta JR Prince R Gaich GA Reginster JY. Effect of parathyroid hormone (1–34) on fractures and bone mineral density in postmenopausal women with osteoporosis. New Engl J Med. (2001) 344:1434–41. doi: 10.1056/nejm200105103441904 11346808

[33] Leidi M Dellera F Mariotti M Maier JA. High magnesium inhibits human osteoblast differentiation *in vitro* . Magnesium Res. (2011) 24:1–6. doi: 10.1684/mrh.2011.0271 21421455

[34] Pironi L Malucelli E Guidetti M Lanzoni E Farruggia G Pinna AD. The complex relationship between magnesium and serum parathyroid hormone: A study in patients with chronic intestinal failure. Magnesium Res. (2009) 22:37–43. 19441273

[35] Ohya M Negi S Sakaguchi T Koiwa F Ando R Komatsu Y. Significance of serum magnesium as an independent correlative factor on the parathyroid hormone level in uremic patients. J Clin Endocrinol Metab. (2014) 99:3873–8. doi: 10.1210/jc.2013-4396 24937533

[36] Cheung MM Dall RD Shewokis PA Altasan A Volpe SL Amori R. The effect of combined magnesium and vitamin D supplementation on vitamin D status, systemic inflammation, and blood pressure: A randomized double-blinded controlled trial. Nutr (Burbank Los Angeles County Calif). (2022) 99–100:111674. doi: 10.1016/j.nut.2022.111674 35576873

[37] Rude RK Gruber HE Wei LY Frausto A Mills BG. Magnesium deficiency: effect on bone and mineral metabolism in the mouse. Calcified Tissue Int. (2003) 72:32–41. doi: 10.1007/s00223-001-1091-1 12370796

[38] Mazur A Maier JA Rock E Gueux E Nowacki W Rayssiguier Y. Magnesium and the inflammatory response: potential physiopathological implications. Arch Biochem biophysics. (2007) 458:48–56. doi: 10.1016/j.abb.2006.03.031 16712775

[39] Baker-LePain JC Nakamura MC Lane NE. Effects of inflammation on bone: an update. Curr Opin Rheumatol. (2011) 23:389–95. doi: 10.1097/BOR.0b013e3283474dbe 21532485

[40] Ding WG Yan WH Wei ZX Liu JB. Difference in intraosseous blood vessel volume and number in osteoporotic model mice induced by spinal cord injury and sciatic nerve resection. J Bone mineral Metab. (2012) 30:400–7. doi: 10.1007/s00774-011-0328-y 22065237

[41] Prisby RD Ramsey MW Behnke BJ Dominguez JM 2nd Donato AJ Allen MR. Aging reduces skeletal blood flow, endothelium-dependent vasodilation, and no bioavailability in rats. J Bone mineral research: Off J Am Soc Bone Mineral Res. (2007) 22:1280–8. doi: 10.1359/jbmr.070415 17451371

[42] Yang C Yuan G Zhang J Tang Z Zhang X Dai K. Effects of magnesium alloys extracts on adult human bone marrow-derived stromal cell viability and osteogenic differentiation. Biomed materials (Bristol England). (2010) 5:45005. doi: 10.1088/1748-6041/5/4/045005 20571183

[43] He LY Zhang XM Liu B Tian Y Ma WH. Effect of magnesium ion on human osteoblast activity. Braz J Med Biol Res = Rev Bras pesquisas medicas e biologicas. (2016) 49. doi: 10.1590/1414-431x20165257 PMC4942226 27383121

[44] Abed E Moreau R. Importance of melastatin-like transient receptor potential 7 and magnesium in the stimulation of osteoblast proliferation and migration by platelet-derived growth factor. Am J Physiol Cell Physiol. (2009) 297:C360–8. doi: 10.1152/ajpcell.00614.2008 19474290

[45] Brown EM MacLeod RJ. Extracellular calcium sensing and extracellular calcium signaling. Physiol Rev. (2001) 81:239–97. doi: 10.1152/physrev.2001.81.1.239 11152759

[46] Ferment O Garnier PE Touitou Y. Comparison of the feedback effect of magnesium and calcium on parathyroid hormone secretion in man. J Endocrinol. (1987) 113:117–22. doi: 10.1677/joe.0.1130117 3585222

[47] Brown AJ Zhong M Ritter C Brown EM Slatopolsky E. Loss of calcium responsiveness in cultured bovine parathyroid cells is associated with decreased calcium receptor expression. Biochem Biophys Res Commun. (1995) 212:861–7. doi: 10.1006/bbrc.1995.2048 7626122

[48] Bellido T Ali AA Gubrij I Plotkin LI Fu Q O'Brien CA. Chronic elevation of parathyroid hormone in mice reduces expression of sclerostin by osteocytes: A novel mechanism for hormonal control of osteoblastogenesis. Endocrinology. (2005) 146:4577–83. doi: 10.1210/en.2005-0239 16081646

[49] Mahalingam CD Sampathi BR Sharma S Datta T Das V Abou-Samra AB. Mkp1-dependent pth modulation of bone matrix mineralization in female mice is osteoblast maturation stage specific and involves P-erk and P-P38 mapks. J Endocrinol. (2013) 216:315–29. doi: 10.1530/joe-12-0372 PMC3796767 23197743

[50] Chen T Wang Y Hao Z Hu Y Li J. Parathyroid hormone and its related peptides in bone metabolism. Biochem Pharmacol. (2021) 192:114669. doi: 10.1016/j.bcp.2021.114669 34224692

[51] Jilka RL. Molecular and cellular mechanisms of the anabolic effect of intermittent pth. Bone. (2007) 40:1434–46. doi: 10.1016/j.bone.2007.03.017 PMC1995599 17517365

[52] Hu G Yu Y Sharma D Pruett-Miller SM Ren Y Zhang GF. Glutathione limits runx2 oxidation and degradation to regulate bone formation. JCI Insight. (2023) 8. doi: 10.1172/jci.insight.166888 PMC10543723 37432749

[53] Allan EH Häusler KD Wei T Gooi JH Quinn JM Crimeen-Irwin B. Ephrinb2 regulation by pth and pthrp revealed by molecular profiling in differentiating osteoblasts. J Bone mineral research: Off J Am Soc Bone Mineral Res. (2008) 23:1170–81. doi: 10.1359/jbmr.080324 18627264

[54] Pasquale EB. Eph-ephrin bidirectional signaling in physiology and disease. Cell. (2008) 133:38–52. doi: 10.1016/j.cell.2008.03.011 18394988

[55] Takyar FM Tonna S Ho PW Crimeen-Irwin B Baker EK Martin TJ. Ephrinb2/ephb4 inhibition in the osteoblast lineage modifies the anabolic response to parathyroid hormone. J Bone mineral research: Off J Am Soc Bone Mineral Res. (2013) 28:912–25. doi: 10.1002/jbmr.1820 23165727

[56] Bikle DD Sakata T Leary C Elalieh H Ginzinger D Rosen CJ. Insulin-like growth factor I is required for the anabolic actions of parathyroid hormone on mouse bone. J Bone mineral research: Off J Am Soc Bone Mineral Res. (2002) 17:1570–8. doi: 10.1359/jbmr.2002.17.9.1570 12211426

[57] Shimizu E Selvamurugan N Westendorf JJ Olson EN Partridge NC. Hdac4 represses matrix metalloproteinase-13 transcription in osteoblastic cells, and parathyroid hormone controls this repression. J Biol Chem. (2010) 285:9616–26. doi: 10.1074/jbc.M109.094862 PMC2843211 20097749

[58] Baron R Kneissel M. Wnt signaling in bone homeostasis and disease: from human mutations to treatments. Nat Med. (2013) 19:179–92. doi: 10.1038/nm.3074 23389618

[59] Krishnan V Bryant HU Macdougald OA. Regulation of bone mass by wnt signaling. J Clin Invest. (2006) 116:1202–9. doi: 10.1172/jci28551 PMC1451219 16670761

[60] Kato M Patel MS Levasseur R Lobov I Chang BH Glass DA 2nd. Cbfa1-independent decrease in osteoblast proliferation, osteopenia, and persistent embryonic eye vascularization in mice deficient in lrp5, a wnt coreceptor. J Cell Biol. (2002) 157:303–14. doi: 10.1083/jcb.200201089 PMC2199263 11956231

[61] Glass DA 2nd Bialek P Ahn JD Starbuck M Patel MS Clevers H. Canonical wnt signaling in differentiated osteoblasts controls osteoclast differentiation. Dev Cell. (2005) 8:751–64. doi: 10.1016/j.devcel.2005.02.017 15866165

[62] Lawal RA Zhou X Batey K Hoffman CM Georger MA Radtke F. The notch ligand jagged1 regulates the osteoblastic lineage by maintaining the osteoprogenitor pool. J Bone mineral research: Off J Am Soc Bone Mineral Res. (2017) 32:1320–31. doi: 10.1002/jbmr.3106 PMC5466455 28277610

[63] Delgado-Calle J McAndrews K Wu G Orr AL Ferrari A Tu X. The notch pathway regulates the bone gain induced by pth anabolic signaling. FASEB journal: Off Publ Fed Am Societies Exp Biol. (2022) 36:e22196. doi: 10.1096/fj.202101807R PMC8855690 35137455

[64] Canalis E Schilling L Yee SP Lee SK Zanotti S. Hajdu cheney mouse mutants exhibit osteopenia, increased osteoclastogenesis, and bone resorption. J Biol Chem. (2016) 291:1538–51. doi: 10.1074/jbc.M115.685453 PMC4722436 26627824

[65] Yu J Schilling L Eller T Canalis E. Hairy and enhancer of split 1 is a primary effector of notch2 signaling and induces osteoclast differentiation and function. J Biol Chem. (2021) 297:101376. doi: 10.1016/j.jbc.2021.101376 34742737 PMC8633688

[66] Kode A Manavalan JS Mosialou I Bhagat G Rathinam CV Luo N. Leukaemogenesis induced by an activating B-catenin mutation in osteoblasts. Nature. (2014) 506:240–4. doi: 10.1038/nature12883 PMC4116754 24429522

[67] Tu X Delgado-Calle J Condon KW Maycas M Zhang H Carlesso N. Osteocytes mediate the anabolic actions of canonical wnt/B-catenin signaling in bone. Proc Natl Acad Sci United States America. (2015) 112:E478–86. doi: 10.1073/pnas.1409857112 PMC4321271 25605937

[68] Atkins GJ Anderson PH Findlay DM Welldon KJ Vincent C Zannettino AC. Metabolism of vitamin D3 in human osteoblasts: evidence for autocrine and paracrine activities of 1 alpha,25-dihydroxyvitamin D3. Bone. (2007) 40:1517–28. doi: 10.1016/j.bone.2007.02.024 17395559

[69] Döhla J Kuuluvainen E Gebert N Amaral A Englund JI Gopalakrishnan S. Metabolic determination of cell fate through selective inheritance of mitochondria. Nat Cell Biol. (2022) 24:148–54. doi: 10.1038/s41556-021-00837-0 PMC7612378 35165416

[70] Maehata Y Takamizawa S Ozawa S Kato Y Sato S Kubota E. Both direct and collagen-mediated signals are required for active vitamin D3-elicited differentiation of human osteoblastic cells: roles of osterix, an osteoblast-related transcription factor. Matrix biology: J Int Soc Matrix Biol. (2006) 25:47–58. doi: 10.1016/j.matbio.2005.09.001 16266799

[71] Wang D Song J Ma H. An *in vitro* experimental insight into the osteoblast responses to vitamin D3 and its metabolites. Pharmacology. (2018) 101:225–35. doi: 10.1159/000486446 29393236

[72] van Leeuwen JP van Driel M van den Bemd GJ Pols HA. Vitamin D control of osteoblast function and bone extracellular matrix mineralization. Crit Rev eukaryotic Gene Expression. (2001) 11:199–226. doi: 10.1615/CritRevEukarGeneExpr.v11.i1-3 11693961

[73] Cui J Ma C Qiu J Ma X Wang X Chen H. A novel interaction between insulin-like growth factor binding protein-6 and the vitamin D receptor inhibits the role of vitamin D3 in osteoblast differentiation. Mol Cell Endocrinol. (2011) 338:84–92. doi: 10.1016/j.mce.2011.03.011 21458526

[74] Fretz JA Zella LA Kim S Shevde NK Pike JW. 1,25-dihydroxyvitamin D3 induces expression of the wnt signaling co-regulator lrp5 via regulatory elements located significantly downstream of the gene's transcriptional start site. J Steroid Biochem Mol Biol. (2007) 103:440–5. doi: 10.1016/j.jsbmb.2006.11.018 PMC1868540 17229572

[75] Guler E Baripoglu YE Alenezi H Arikan A Babazade R Unal S. Vitamin D(3)/vitamin K(2)/magnesium-loaded polylactic acid/tricalcium phosphate/polycaprolactone composite nanofibers demonstrated osteoinductive effect by increasing runx2 via wnt/B-catenin pathway. Int J Biol macromolecules. (2021) 190:244–58. doi: 10.1016/j.ijbiomac.2021.08.196 34492244

[76] Swaminathan R. Magnesium metabolism and its disorders. Clin biochemist Rev. (2003) 24:47–66. PMC1855626 18568054

[77] Kunutsor SK Whitehouse MR Blom AW Laukkanen JA. Low serum magnesium levels are associated with increased risk of fractures: A long-term prospective cohort study. Eur J Epidemiol. (2017) 32:593–603. doi: 10.1007/s10654-017-0242-2 28405867 PMC5570773

[78] Chambers TJ Fuller K McSheehy PM Pringle JA. The effects of calcium regulating hormones on bone resorption by isolated human osteoclastoma cells. J Pathol. (1985) 145:297–305. doi: 10.1002/path.1711450403 2987469

[79] Kaji H Sugimoto T Kanatani M Fukase M Chihara K. Involvement of dual signal transduction systems in the stimulation of osteoclast-like cell formation by parathyroid hormone and parathyroid hormone-related peptide. Biochem Biophys Res Commun. (1993) 194:157–62. doi: 10.1006/bbrc.1993.1798 8392835

[80] Ma YL Cain RL Halladay DL Yang X Zeng Q Miles RR. Catabolic effects of continuous human pth (1–38) *in vivo* is associated with sustained stimulation of rankl and inhibition of osteoprotegerin and gene-associated bone formation. Endocrinology. (2001) 142:4047–54. doi: 10.1210/endo.142.9.8356 11517184

[81] Kanzawa M Sugimoto T Kanatani M Chihara K. Involvement of osteoprotegerin/osteoclastogenesis inhibitory factor in the stimulation of osteoclast formation by parathyroid hormone in mouse bone cells. Eur J Endocrinol. (2000) 142:661–4. doi: 10.1530/eje.0.1420661 10822231

[82] Kearns AE Khosla S Kostenuik PJ. Receptor activator of nuclear factor kappab ligand and osteoprotegerin regulation of bone remodeling in health and disease. Endocrine Rev. (2008) 29:155–92. doi: 10.1210/er.2007-0014 PMC2528846 18057140

[83] Huang JC Sakata T Pfleger LL Bencsik M Halloran BP Bikle DD. Pth differentially regulates expression of rankl and opg. J Bone mineral research: Off J Am Soc Bone Mineral Res. (2004) 19:235–44. doi: 10.1359/jbmr.0301226 14969393

[84] Fu Q Jilka RL Manolagas SC O'Brien CA. Parathyroid hormone stimulates receptor activator of nfkappa B ligand and inhibits osteoprotegerin expression via protein kinase a activation of camp-response element-binding protein. J Biol Chem. (2002) 277:48868–75. doi: 10.1074/jbc.M208494200 12364326

[85] Mammoli F Castiglioni S Parenti S Cappadone C Farruggia G Iotti S. Magnesium is a key regulator of the balance between osteoclast and osteoblast differentiation in the presence of vitamin D₃. Int J Mol Sci. (2019) 20. doi: 10.3390/ijms20020385 PMC6358963 30658432

[86] Nieves JW. Bone. Maximizing bone health–magnesium, bmd and fractures. . Nat Rev Endocrinol. (2014) 10:255–6. doi: 10.1038/nrendo.2014.39 24686202

[87] Gu J Zhang X Zhang C Li Y Bian J Liu X. Galectin-3 contributes to the inhibitory effect of Lα,25-(Oh)(2)D(3) on osteoclastogenesis. Int J Mol Sci. (2021) 22. doi: 10.3390/ijms222413334 PMC8708238 34948130

[88] Sakai S Takaishi H Matsuzaki K Kaneko H Furukawa M Miyauchi Y. 1-alpha, 25-dihydroxy vitamin D3 inhibits osteoclastogenesis through ifn-beta-dependent nfatc1 suppression. J Bone mineral Metab. (2009) 27:643–52. doi: 10.1007/s00774-009-0084-4 19449179

[89] Kikuta J Kawamura S Okiji F Shirazaki M Sakai S Saito H. Sphingosine-1-phosphate-mediated osteoclast precursor monocyte migration is a critical point of control in antibone-resorptive action of active vitamin D. Proc Natl Acad Sci United States America. (2013) 110:7009–13. doi: 10.1073/pnas.1218799110 PMC3637769 23569273

[90] Wein MN. Parathyroid hormone signaling in osteocytes. JBMR plus. (2018) 2:22–30. doi: 10.1002/jbm4.10021 30283888 PMC6124166

[91] Wein MN Kronenberg HM. Regulation of bone remodeling by parathyroid hormone. Cold Spring Harbor Perspect Med. (2018) 8. doi: 10.1101/cshperspect.a031237 PMC6071549 29358318

[92] Ben-awadh AN Delgado-Calle J Tu X Kuhlenschmidt K Allen MR Plotkin LI. Parathyroid hormone receptor signaling induces bone resorption in the adult skeleton by directly regulating the rankl gene in osteocytes. Endocrinology. (2014) 155:2797–809. doi: 10.1210/en.2014-1046 PMC4098003 24877630

[93] Deng X Song Y Manson JE Signorello LB Zhang SM Shrubsole MJ. Magnesium, vitamin D status and mortality: results from us national health and nutrition examination survey (Nhanes) 2001 to 2006 and nhanes iii. BMC Med. (2013) 11:187. doi: 10.1186/1741-7015-11-187 23981518 PMC3765911

[94] Dai Q Zhu X Manson JE Song Y Li X Franke AA. Magnesium status and supplementation influence vitamin D status and metabolism: results from a randomized trial. Am J Clin Nutr. (2018) 108:1249–58. doi: 10.1093/ajcn/nqy274 PMC6693398 30541089

[95] Rude RK Adams JS Ryzen E Endres DB Niimi H Horst RL. Low serum concentrations of 1,25-dihydroxyvitamin D in human magnesium deficiency. J Clin Endocrinol Metab. (1985) 61:933–40. doi: 10.1210/jcem-61-5-933 3840173

[96] Sahota O Mundey MK San P Godber IM Hosking DJ. Vitamin D insufficiency and the blunted pth response in established osteoporosis: the role of magnesium deficiency. Osteoporosis international: J established as result cooperation between Eur Foundation Osteoporosis Natl Osteoporosis Foundation USA. (2006) 17:1013–21. doi: 10.1007/s00198-006-0084-3 16596461

[97] Pereira RC Salusky IB Bowen RE Freymiller EG Wesseling-Perry K. Vitamin D sterols increase fgf23 expression by stimulating osteoblast and osteocyte maturation in ckd bone. Bone. (2019) 127:626–34. doi: 10.1016/j.bone.2019.07.026 PMC6715148 31377240

[98] Bergwitz C Jüppner H. Regulation of phosphate homeostasis by pth, vitamin D, and fgf23. Annu Rev Med. (2010) 61:91–104. doi: 10.1146/annurev.med.051308.111339 20059333 PMC4777331

[99] Prisby R Guignandon A Vanden-Bossche A Mac-Way F Linossier MT Thomas M. Intermittent pth(1–84) is osteoanabolic but not osteoangiogenic and relocates bone marrow blood vessels closer to bone-forming sites. J Bone mineral research: Off J Am Soc Bone Mineral Res. (2011) 26:2583–96. doi: 10.1002/jbmr.459 21713994

[100] Kusumbe AP Ramasamy SK Adams RH. Coupling of angiogenesis and osteogenesis by a specific vessel subtype in bone. Nature. (2014) 507:323–8. doi: 10.1038/nature13145 PMC4943525 24646994

[101] Fu R Lv WC Xu Y Gong MY Chen XJ Jiang N. Endothelial zeb1 promotes angiogenesis-dependent bone formation and reverses osteoporosis. Nat Commun. (2020) 11:460. doi: 10.1038/s41467-019-14076-3 31974363 PMC6978338

[102] Mutnuri S Fernandez I Kochar T. Suppression of parathyroid hormone in a patient with severe magnesium depletion. Case Rep Nephrol. (2016) 2016:2608538. doi: 10.1155/2016/2608538 27190662 PMC4850250

[103] Kallio PJ Okamoto K O'Brien S Carrero P Makino Y Tanaka H. Signal transduction in hypoxic cells: inducible nuclear translocation and recruitment of the cbp/P300 coactivator by the hypoxia-inducible factor-1alpha. EMBO J. (1998) 17:6573–86. doi: 10.1093/emboj/17.22.6573 PMC1171004 9822602

[104] Liu Y Berendsen AD Jia S Lotinun S Baron R Ferrara N. Intracellular vegf regulates the balance between osteoblast and adipocyte differentiation. J Clin Invest. (2012) 122:3101–13. doi: 10.1172/jci61209 PMC3428080 22886301

[105] Esbrit P Alvarez-Arroyo MV DE Miguel F Martin O Martinez ME Caramelo C. C-terminal parathyroid hormone-related protein increases vascular endothelial growth factor in human osteoblastic cells. J Am Soc Nephrology: JASN. (2000) 11:1085–92. doi: 10.1681/asn.V1161085 10820172

[106] Mayr-Wohlfart U Waltenberger J Hausser H Kessler S Günther KP Dehio C. Vascular endothelial growth factor stimulates chemotactic migration of primary human osteoblasts. Bone. (2002) 30:472–7. doi: 10.1016/s8756-3282(01)00690-1 11882460

[107] Street J Bao M deGuzman L Bunting S Peale FV Jr. Ferrara N. Vascular endothelial growth factor stimulates bone repair by promoting angiogenesis and bone turnover. Proc Natl Acad Sci United States America. (2002) 99:9656–61. doi: 10.1073/pnas.152324099 PMC124965 12118119

[108] Ding Q Sun P Zhou H Wan B Yin J Huang Y. Lack of endogenous parathyroid hormone delays fracture healing by inhibiting vascular endothelial growth factor−Mediated angiogenesis. Int J Mol Med. (2018) 42:171–81. doi: 10.3892/ijmm.2018.3614 PMC5979887 29620150

[109] Gambardella J De Rosa M Sorriento D Prevete N Fiordelisi A Ciccarelli M. Parathyroid hormone causes endothelial dysfunction by inducing mitochondrial ros and specific oxidative signal transduction modifications. Oxid Med Cell Longevity. (2018) 2018:9582319. doi: 10.1155/2018/9582319 PMC6313989 30662585

[110] Capozzi A Scambia G Lello S. Calcium, vitamin D, vitamin K2, and magnesium supplementation and skeletal health. Maturitas. (2020) 140:55–63. doi: 10.1016/j.maturitas.2020.05.020 32972636

[111] Scaturro D Vitagliani F Terrana P Tomasello S Camarda L Letizia Mauro G. Does the association of therapeutic exercise and supplementation with sucrosomial magnesium improve posture and balance and prevent the risk of new falls? Aging Clin Exp Res. (2022) 34:545–53. doi: 10.1007/s40520-021-01977-x PMC8894156 34510395

[112] Wang Q Si K Xing X Ye X Liu Z Chen J. Association between dietary magnesium intake and muscle mass among hypertensive population: evidence from the national health and nutrition examination survey. Nutr J. (2024) 23:37. doi: 10.1186/s12937-024-00940-6 38509619 PMC10956219

[113] Zhang Y Xun P Wang R Mao L He K. Can magnesium enhance exercise performance? Nutrients. (2017) 9:946. doi: 10.3390/nu9090946 28846654 PMC5622706

[114] Dørup I Clausen T. Effects of magnesium and zinc deficiencies on growth and protein synthesis in skeletal muscle and the heart. Br J Nutr. (1991) 66:493–504. doi: 10.1079/bjn19910050 1772873

[115] Bano G Trevisan C Carraro S Solmi M Luchini C Stubbs B. Inflammation and sarcopenia: A systematic review and meta-analysis. Maturitas. (2017) 96:10–5. doi: 10.1016/j.maturitas.2016.11.006 28041587

[116] López-Baltanás R Encarnación Rodríguez-Ortiz M Canalejo A Díaz-Tocados JM Herencia C Leiva-Cepas F. Magnesium supplementation reduces inflammation in rats with induced chronic kidney disease. Eur J Clin Invest. (2021) 51:e13561. doi: 10.1111/eci.13561 33870500

[117] Simental-Mendia LE Sahebkar A Rodriguez-Moran M Zambrano-Galvan G Guerrero-Romero F. Effect of magnesium supplementation on plasma C-reactive protein concentrations: A systematic review and meta-analysis of randomized controlled trials. Curr Pharm design. (2017) 23:4678–86. doi: 10.2174/1381612823666170525153605 28545353

[118] Bodine SC Stitt TN Gonzalez M Kline WO Stover GL Bauerlein R. Akt/mtor pathway is a crucial regulator of skeletal muscle hypertrophy and can prevent muscle atrophy *in vivo* . Nat Cell Biol. (2001) 3:1014–9. doi: 10.1038/ncb1101-1014 11715023

[119] Jagoe RT Goldberg AL. What do we really know about the ubiquitin-proteasome pathway in muscle atrophy? Curr Opin Clin Nutr Metab Care. (2001) 4:183–90. doi: 10.1097/00075197-200105000-00003 11517350

[120] Lecker SH Jagoe RT Gilbert A Gomes M Baracos V Bailey J. Multiple types of skeletal muscle atrophy involve a common program of changes in gene expression. FASEB journal: Off Publ Fed Am Societies Exp Biol. (2004) 18:39–51. doi: 10.1096/fj.03-0610com 14718385

[121] Dominguez LJ Barbagallo M Lauretani F Bandinelli S Bos A Corsi AM. Magnesium and muscle performance in older persons: the inchianti study. Am J Clin Nutr. (2006) 84:419–26. doi: 10.1093/ajcn/84.1.419 PMC2669297 16895893

[122] Cui CY Driscoll RK Piao Y Chia CW Gorospe M Ferrucci L. Skewed macrophage polarization in aging skeletal muscle. Aging Cell. (2019) 18:e13032. doi: 10.1111/acel.13032 31478346 PMC6826159

[123] Wang Y Wehling-Henricks M Welc SS Fisher AL Zuo Q Tidball JG. Aging of the immune system causes reductions in muscle stem cell populations, promotes their shift to a fibrogenic phenotype, and modulates sarcopenia. FASEB journal: Off Publ Fed Am Societies Exp Biol. (2019) 33:1415–27. doi: 10.1096/fj.201800973R PMC6355087 30130434

[124] Cui C Bao Z Chow SK Wong RMY Welch A Qin L. Coapplication of magnesium supplementation and vibration modulate macrophage polarization to attenuate sarcopenic muscle atrophy through pi3k/akt/mtor signaling pathway. Int J Mol Sci. (2022) 23:12944. doi: 10.3390/ijms232112944 36361730 PMC9654727

[125] Farsinejad-Marj M Saneei P Esmaillzadeh A. Dietary magnesium intake, bone mineral density and risk of fracture: A systematic review and meta-analysis. Osteoporosis international: J established as result cooperation between Eur Foundation Osteoporosis Natl Osteoporosis Foundation USA. (2016) 27:1389–99. doi: 10.1007/s00198-015-3400-y 26556742

[126] Groenendijk I van Delft M Versloot P van Loon LJC de Groot LCPGM. Impact of magnesium on bone health in older adults: A systematic review and meta-analysis. Bone. (2022) 154:116233. doi: 10.1016/j.bone.2021.116233 34666201

[127] Aydin H Deyneli O Yavuz D Gözü H Mutlu N Kaygusuz I. Short-term oral magnesium supplementation suppresses bone turnover in postmenopausal osteoporotic women. Biol Trace element Res. (2010) 133:136–43. doi: 10.1007/s12011-009-8416-8 19488681

[128] Stendig-Lindberg G Tepper R Leichter I. Trabecular bone density in a two year controlled trial of peroral magnesium in osteoporosis. Magnesium Res. (1993) 6:155–63. 8274361

[129] Fouhy LE Mangano KM Zhang X Hughes BD Tucker KL Noel SE. Association between a calcium-to-magnesium ratio and osteoporosis among Puerto Rican adults. J Nutr. (2023) 153:2642–50. doi: 10.1016/j.tjnut.2023.05.009 PMC10550845 37164266

[130] Veronese N Stubbs B Solmi M Noale M Vaona A Demurtas J. Dietary magnesium intake and fracture risk: data from a large prospective study. Br J Nutr. (2017) 117:1570–6. doi: 10.1017/s0007114517001350 PMC5753403 28631583

[131] Ryder KM Shorr RI Bush AJ Kritchevsky SB Harris T Stone K. Magnesium intake from food and supplements is associated with bone mineral density in healthy older white subjects. J Am Geriatrics Soc. (2005) 53:1875–80. doi: 10.1111/j.1532-5415.2005.53561.x 16274367

[132] Abraham GE Grewal H. A total dietary program emphasizing magnesium instead of calcium. Effect on the mineral density of calcaneous bone in postmenopausal women on hormonal therapy. J Reprod Med. (1990) 35:503–7. 2352244

[133] Groenendijk I den Boeft L van Loon LJC de Groot L. High versus low dietary protein intake and bone health in older adults: A systematic review and meta-analysis. Comput Struct Biotechnol J. (2019) 17:1101–12. doi: 10.1016/j.csbj.2019.07.005 PMC6704341 31462966

[134] Rizzoli R Stevenson JC Bauer JM van Loon LJ Walrand S Kanis JA. The role of dietary protein and vitamin D in maintaining musculoskeletal health in postmenopausal women: A consensus statement from the European society for clinical and economic aspects of osteoporosis and osteoarthritis (Esceo). Maturitas. (2014) 79:122–32. doi: 10.1016/j.maturitas.2014.07.005 25082206

[135] Gregson CL Armstrong DJ Bowden J Cooper C Edwards J Gittoes NJL. Uk clinical guideline for the prevention and treatment of osteoporosis. Arch osteoporosis. (2022) 17:58. doi: 10.1007/s11657-022-01061-5 PMC8979902 35378630

[136] Kristjansdottir AG Johannsson E Thorsdottir I. Effects of a school-based intervention on adherence of 7–9-year-olds to food-based dietary guidelines and intake of nutrients. Public Health Nutr. (2010) 13:1151–61. doi: 10.1017/s1368980010000716 20409359

[137] Groenendijk I Kramer CS den Boeft LM Hobbelen HSM van der Putten GJ de Groot L. Hip fracture patients in geriatric rehabilitation show poor nutritional status, dietary intake and muscle health. Nutrients. (2020) 12. doi: 10.3390/nu12092528 PMC7551784 32825439

[138] Verlaan S Aspray TJ Bauer JM Cederholm T Hemsworth J Hill TR. Nutritional status, body composition, and quality of life in community-dwelling sarcopenic and non-sarcopenic older adults: A case-control study. Clin Nutr (Edinburgh Scotland). (2017) 36:267–74. doi: 10.1016/j.clnu.2015.11.013 26689868

